# An image retrieval framework for real-time endoscopic image retargeting

**DOI:** 10.1007/s11548-017-1620-7

**Published:** 2017-06-02

**Authors:** Menglong Ye, Edward Johns, Benjamin Walter, Alexander Meining, Guang-Zhong Yang

**Affiliations:** 1 The Hamlyn Centre for Robotic Surgery, Imperial College London, London, UK; 2 Dyson Robotics Laboratory, Imperial College London, London, UK; 3 Centre of Internal Medicine, Ulm University, Ulm, Germany

**Keywords:** Endoscopic navigation, Retargeting, Image recognition, Binary codes

## Abstract

**Purpose:**

Serial endoscopic examinations of a patient are important for early diagnosis of malignancies in the gastrointestinal tract. However, retargeting for optical biopsy is challenging due to extensive tissue variations between examinations, requiring the method to be tolerant to these changes whilst enabling real-time retargeting.

**Method:**

This work presents an image retrieval framework for inter-examination retargeting. We propose both a novel image descriptor tolerant of long-term tissue changes and a novel descriptor matching method in real time. The descriptor is based on histograms generated from regional intensity comparisons over multiple scales, offering stability over long-term appearance changes at the higher levels, whilst remaining discriminative at the lower levels. The matching method then learns a hashing function using random forests, to compress the string and allow for fast image comparison by a simple Hamming distance metric.

**Results:**

A dataset that contains 13 in vivo gastrointestinal videos was collected from six patients, representing serial examinations of each patient, which includes videos captured with significant time intervals. Precision-recall for retargeting shows that our new descriptor outperforms a number of alternative descriptors, whilst our hashing method outperforms a number of alternative hashing approaches.

**Conclusion:**

We have proposed a novel framework for optical biopsy in serial endoscopic examinations. A new descriptor, combined with a novel hashing method, achieves state-of-the-art retargeting, with validation on in vivo videos from six patients. Real-time performance also allows for practical integration without disturbing the existing clinical workflow.

## Introduction

Endoscopic examinations have been widely used for visualising the human gastrointestinal (GI) tract. Surveillance endoscopy has been a popular approach for monitoring abnormal changes, such as colorectal polyps and Barretts’ esophagus. A typical endoscopic procedure involves taking tissue samples for histological analysis afterwards, which is both time-consuming and expensive. With the advances in biophotonics, optical biopsy has emerged as a technique for providing in vivo, in situ, and real-time tissue characterisation, such that in time, curative treatment can be performed. Techniques for optical biopsy include narrow band imaging (NBI), blue light imaging (BLI), and confocal laser endomicroscopy (CLE), which can be either integrated into endoscope systems, or manufactured as an external probe-based device, to retrieve the cellular details on the tissue.

**Fig. 1:**
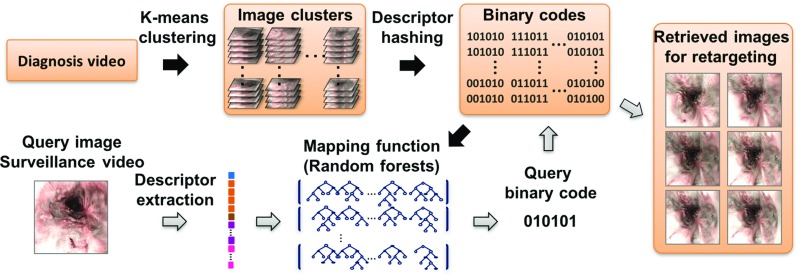
An overview of the proposed image retrieval framework for inter-examination retargeting. *Black arrows* indicate the training phase that hashes the descriptors and learns the encoding function, whilst *grey arrows* indicate the retargeting phase that retrieves relevant images to a query image

Despite the advantages provided by optical biopsy, retargeting of a biopsied location remains a challenging problem for both intra- and inter-examination. In [1], a feature matching method based on Markov random fields was proposed for intra-examination retargeting. Allain et al. [2] combined feature matching with epipolar geometry to provide biopsied location estimation with an uncertainty score. Alternatively, a 3D tracking approach was introduced by Mountney et al. [3] that uses simultaneous localisation and mapping (SLAM) to achieve consistent retargeting in a relatively static endoscopic environment. In [4–6], retargeting of a biopsied location was formulated as a 2D object tracking task, where detectors based on random forests were included to learn online the appearance of the biopsied area. Later, a hybrid approach dealing with occlusion was proposed by Mouton et al. [7] to perform efficient retargeting during probe-based CLE examinations. However, the above approaches would encounter difficulties when applied to serial examinations where there is long-term variation in local tissue appearance.

For retargeting over successive examinations of a patient, which we refer to as the inter-examination retargeting problem, endoscopic video manifolds (EVM) was proposed by Atasoy et al. [8], to learn a low-dimensional intrinsic representation of the video collected in the first examination. This mapping was then learned based on locality preserving projections [9], such that retargeting of a query image in the second examination can be achieved via image retrieval. In [10], a detailed study was performed to evaluate visual descriptors used for viewpoint selection in endoscopic surveillance. In addition to vision-based approaches, the use of external positioning sensors has also been considered. In [11], multiple electromagnetic sensors were used to register the trajectories of the endoscope motion across examinations. Although this method is not affected by the issues of image-based inter-examination retargeting, addition of extra sensors could introduce further complexity to the setup.

Our recent work in [12] introduced a vision-based framework for inter-examination retargeting to assist optical biopsy procedures. The proposed framework (see Fig. 1) formulates retargeting as an image retrieval task to enable retargeting of biopsied locations in the second (surveillance) examination based on the targets recorded in the first (diagnosis) examination. A global image description scheme is designed by pooling the spatial information obtained from regional comparisons over multiple scales. Inspired by hashing-based techniques, the global descriptors are compressed into short binary strings with a novel random forest-based encoding function. This then enables real-time retargeting, without interfering with the current clinical workflow. Following our previous work, this paper provides extended descriptions of the methodology, as well as new insights into the technical contributions. Furthermore, other alternative approaches are added into our comparison studies with further validation on in vivo GI video sequences collected from six patients.

## Methods

### A multi-level endoscopic image descriptor

Over the last two decades, there has been significant progress in using keypoint-based approaches for image description. One of these is the bag-of-words (BOW) framework [13], which builds a dictionary by performing clustering on local features, such as SIFT [14]. A descriptor of an image is obtained by extracting these features and collecting a frequency histogram from individual words (in the dictionary) for this image. Recently, BOW has been combined with geometric constraints for image retrieval [15] and place recognition [16]. However, the success of these approaches depends on re-occurrences of same local keypoints across different views, which is not always possible for endoscopic scenes as these typically undergo long-term appearance changes on the local tissue surface.

**Fig. 2:**
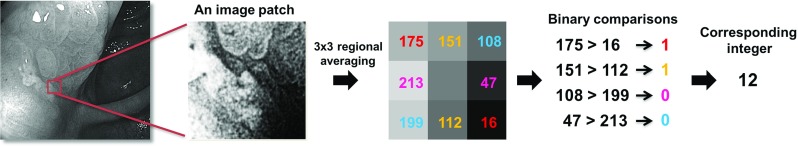
Proposed binary pattern performs regional comparisons to obtain a single integer describing the image location

**Fig. 3:**
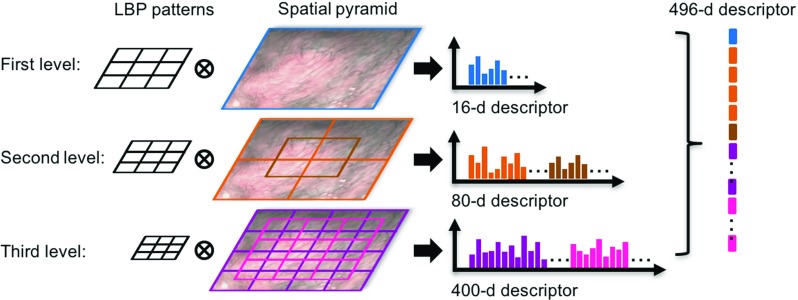
Spatial pyramid pooling is applied to aggregate the responses from regional comparisons at multiple scales, which generates a 496-*d* image descriptor

Recently, descriptors based on local binary patterns (LBPs) have emerged to be powerful tools for scene recognition [17], object tracking [18], and 3D reconstruction [19]. The main advantages of LBPs include the tolerance to illumination changes and the superior computational efficiency. Compared to keypoint-based descriptions, such as BOW, LBPs-based descriptors also do not rely on consistent detection of same keypoints over images, thus providing more robustness to long-term tissue appearance changes.

In this paper, we use a symmetric version of LBP based on regional comparisons Fig. 2a. Our LBP performs 4 diagonal comparisons inside an image patch, yielding a 4-bit binary string for this patch. This binary string is then converted into an integer ranging from 0 to 15. With this, a 16-*dimensional*(*d*) image histogram descriptor can be simply obtained by sliding this pattern over the entire image. To consider the global geometry that would be effective for endoscopic scene description, we employ the spatial pyramid pooling approach [20] to aggregate the responses of LBP across various scales and locations. Here, we use a three-level coarse-to-fine representation, as shown in Fig. 3.

In addition to the first level that produces 16-*d* descriptor, for the second level, the image is divided into $2\times 2$ partitions with an additional partition overlapping at the centre, providing a 80-*d* descriptor. In the third level, we divide the image into $4\times 4$ partitions, with additional $3\times 3$ partitions overlapped, resulting in a 400-*d* descriptor. To balance the contributions from different levels, the LBP masks contain $24\times 24$, $12\times 12$ and $6\times 6$ pixels for the first, second and third levels, respectively. Finally, a 496-*d* global descriptor for this image is obtained by concatenating the descriptors across all levels.

### Compact binary code representation

Let us now denote the video sequences collected in the first (diagnosis) and second (surveillance) examinations as $\mathcal {O}_1$ and $\mathcal {O}_2$, respectively. During the surveillance examination, retargeting of a query image (in $\mathcal {O}_2$) is required to be real time such that a regular clinical procedure would not be interfered with. To enable the real-time retargeting capability, we adopt hashing which has proved to be efficient for large-scale image retrieval [21–24]). We follow the two-step hashing approach in [24] to compress the image descriptors into compact binary codes and then learn the mapping function via a novel random forests hash. This allows for fast matching between descriptors based on Hamming distance computation. Furthermore, a quadratic loss function is used for learning the hashing function that maps the original descriptors to a new space, where images from the same scene have a smaller distance.

In this work, we adopt supervised hashing, requiring a scene label for each image in the training image set. We define a scene as a cluster of adjacent images which represent the same topological location. To obtain the scene labels for images, we perform image clustering on the diagnosis video collected in the first examination similar to [8]. Specifically, we use an semiautomatic approach that performs K-means (intensity-based) clustering, followed by manually merging similar clusters. This results in an affinity matrix $\mathcal {A}$ where $a_{ij} = 1$ if $x_i$ and $x_j$ have the same scene label, and $a_{ij} = 0$ if not.

Given a set of image descriptors extracted from the diagnosis video, which are denoted as $\left\{ \mathbf {x}_{i} \right\} _{i=1}^{n}$, our aim is to infer their corresponding *m*-bit binary codes $\left\{ \mathbf {b}_{i} \right\} _{i=1}^{n}$. This inference is performed by encouraging the Hamming distance between images of the same scene to be small, whilst large for images of different scenes. We sequentially obtain each bit in the binary code by optimising for *r*-th bit with the objective function:

1 $$\begin{aligned} \begin{aligned} \min _{\mathbf {b}_{\left( r\right) }}&\sum _{i=1}^{n}\sum _{j=1}^{n}l_r\left( b_{r,i}, b_{r,j};a_{ij} \right) , \\&\text {s.t.}\, \mathbf {b}_{\left( r\right) }\in \left\{ -1,1 \right\} ^{n} \end{aligned} \end{aligned}$$

where $b_{r,i}$ and $b_{r,j}$ are the *r*-th bits for images *i* and *j*, respectively. Here, $\mathbf {b}_{\left( r\right) }$ represents a vector that concatenates the *r*-th bits of *n* images. Therefore, this optimisation sequentially seeks the values of $\mathbf {b}_{\left( r\right) }$ for each bit.

Following [24], we consider a hash loss function $l\left( b_1, b_2 \right)$ that takes binary variables $b_1, b_2 \in \lbrace -1,1 \rbrace$ as input and satisfies $l\left( 1,1\right) =l\left( -1,-1\right)$ and $l\left( -1,1\right) =l\left( 1,-1\right)$. This loss can be replaced with an equivalent quadratic function defined as:

2 $$\begin{aligned} \begin{aligned} h\left( b_1, b_2 \right)&= \dfrac{1}{2}\left[ b_1 b_2 \left( l^{11}-l^{-11}\right) + l^{11} + l^{-11} \right] \\&= l\left( b_1, b_2 \right) , \end{aligned} \end{aligned}$$

Here, $l^{11}$ and $l^{-11}$ are the constants that represent $l\left( 1,1\right)$ and $l\left( -1,1\right)$, respectively. Note that, Eq. 2 can be proved by checking all the possible binary inputs. For example, when $b_1=b_2=1$, we have

3 $$\begin{aligned} h\left( 1,1 \right) = \left[ l^{11}-l^{-11} + l^{11}+l^{-11}\right] = l\left( 1,1\right) , \end{aligned}$$

and when $b_1=-1$ and $b_2=1$, we can obtain

4 $$\begin{aligned} h\left( -1,1 \right)= & {} \dfrac{1}{2}\left[ -1\cdot 1\cdot \left( l^{11}-l^{-11} \right) + l^{11}+l^{-11}\right] \nonumber \\= & {} l\left( -1,1\right) . \end{aligned}$$

Similar equations can also be derived for $h\left( -1,-1 \right)$ and $h\left( 1,-1 \right)$. Given that $l^{11}+l^{-11}$ results in a constant, we now use Eq. 2 to reformulate Eq. 1 as

5 $$\begin{aligned} \begin{aligned} \min _{\mathbf {b}_{\left( r\right) }}&\sum _{i=1}^{n}\sum _{j=1}^{n} b_{r,i}b_{b,j} \left( l^{11}_{r,i,j}-l^{-11}_{r,i,j}\right) , \\&\text {s.t.}\, \mathbf {b}_{\left( r\right) }\in \left\{ -1,1 \right\} ^{n}. \end{aligned} \end{aligned}$$

When considering the affinity label between images *i* and *j*, we have $l^{11}_{r,i,j}=l_r\left( 1,1;a_{ij}\right)$ and $l^{-11}_{r,i,j}=l_r\left( -1,1;a_{ij}\right)$. Let us denote $c_{r,i,j} = l^{11}_{r,i,j}-l^{-11}_{r,i,j}$, and define matrix $\mathcal {C}$ that contains all the $c_{r,i,j}$ elements. The objective is finally turned into a matrix representation:

6 $$\begin{aligned} \begin{aligned}&\min _{\mathbf {b}_{\left( r\right) }} \mathbf {b}_{\left( r\right) }^{T} \mathcal {C} \mathbf {b}_{\left( r\right) }, \\&\text {s.t.}\, \mathbf {b}_{\left( r\right) }\in \left\{ -1,1 \right\} ^{n}. \end{aligned} \end{aligned}$$

Note that, for solving this unconstrained binary quadratic problem, we perform a series of local optimisations via graph-cut [24]. Furthermore, in this work, we employ a hinge loss function, defined as

7 $$\begin{aligned}&l_r\left( b_{r,i}, b_{r,j};a_{ij}\right) \nonumber \\&\quad = {\left\{ \begin{array}{ll} \left[ 0-\mathcal {D}\left( \mathbf {b}_{i}^r,\mathbf {b}_{j}^r \right) \right] ^{2},&{} \quad \text {if } a_{ij} = 1\\ \left[ \max \left( 0.5m -\mathcal {D}\left( \mathbf {b}_{i}^r, \mathbf {b}_{j}^r \right) ,0\right) \right] ^{2},&{} \quad \text {if } a_{ij} = 0 \end{array}\right. } \end{aligned}$$

where $\mathbf {b}^r_i$ and $\mathbf {b}^r_j$ denote the first *r* bits for $\mathbf {b}_i$ and $\mathbf {b}_j$, respectively. $\mathcal {D}\left( \cdot ,\cdot \right)$ indicates the Hamming distance. Equation 7 encourages the images of same scene to be close and pushes the images of different scenes to have distances larger than half the maximum distance (0.5 m). It is worth noting that during this sequential optimisation, each current bit (*r*-th bit) derivation uses the results of previous bits ($0-(r-1)$-th bits).

### Mapping function learning

After obtaining the binary codes for the training image set ($\mathcal {O}_1$), the next step is to obtain the binary code of a query image in $\mathcal {O}_2$, such that efficient Hamming distance-based matching can be performed. Note that the optimisation with Eq. 6 only aims to infer the binary codes on the training image set. To allow for out-of-sample extension, we need to learn a mapping function. In this work, we propose to use random forests as this mapping.

Given the global image descriptors $\left\{ \mathbf {x}_{i} \right\} _{i=1}^{n}$ and their corresponding binary codes $\left\{ \mathbf {b}_{i} \right\} _{i=1}^{n}$, we now formulate this mapping function as a set of binary classification functions $\left\{ \phi _{i}\left( \mathbf {x} \right) \right\} _{i=1}^{m}$, with each random forest $\phi _{i}\left( \mathbf {x} \right)$ taking the image descriptor as the input, and returning the label $\lbrace -1,1 \rbrace$ for the *i*-th bit, defined as:

8 $$\begin{aligned}&\phi _{i}\left( \mathbf {x} \right) \nonumber \\&\quad =\left\{ \begin{array}{ll} -1&{} \quad \text {if } \frac{1}{K}\sum _{k=1}^{K} \alpha _{k}\left( \mathbf {x} \right) < 0.5\\ 1&{} \quad \text {otherwise} \end{array}\right. \end{aligned}$$

Here, we train *K* decision trees for each *i*-th hash function, and assign $-1$ or 1 by calculating the average responses from all trees. The training input for each tree $\alpha _{k}\left( \mathbf {x} \right)$ is a subset randomly sampled from $\left\{ \mathbf {x}_{i} \right\} _{i=1}^{n}$.

The split function at each tree node is associated with learning two parameters *s* and $\tau$, which performs a comparison on the *s*-th element in $\mathbf {x}_{i}$ with threshold $\tau$. To grow each decision tree, we maximise an information gain to find the optimal parameters that split the input data *X* into left $X_L$ and right $X_R$ subsets. We define this information gain *I* as

9 $$\begin{aligned} I=\pi \left( X \right) -\dfrac{1}{|X|}\sum _{t\in \left\{ L,R \right\} }|X_{t}|\pi \left( X_{t}\right) \end{aligned}$$

Here, we use the Shannon entropy:

10 $$\begin{aligned} \pi \left( X \right) =-\sum _{y\in \left\{ -1,1 \right\} }p_{y}\log \left( p_{y} \right) , \end{aligned}$$

where $p_y$ indicates the fraction of data in *X* assigned to label *y*. We stop growing a tree when the defined maximum depth has been reached, or the value of *I* is below $e^{-10}$.

In this work, we train *m* random forests, acting as the mapping function $\left\{ \phi _{i}\left( \mathbf {x} \right) \right\} _{i=1}^{m}$ with each generating one bit of the binary code according to Eq. 8. During the surveillance examination, retargeting of a query image is achieved by obtaining its binary code (via the mapping function), followed by comparing the Hamming distance to the binary codes $\left\{ \mathbf {b}_{i} \right\} _{i=1}^{n}$ from the previous diagnosis video. Finally, the relevant images of the query image are retrieved.

**Table 1:** Details of the clustered video dataset with their inter-cluster variances (ICV)

	Patient 1		Patient 2		Patient 3		Patient 4		Patient 5		Patient 6		
Video ID	1	2	3	4	5	6	7	8	9	10	11	12	13
Images	1220	1299	868	991	1056	877	1034	1059	543	679	2071	3663	1518
Clusters	19	20	14	16	18	16	17	17	10	12	26	34	21
ICV	$7.7{+}e5$	$9.7{+}e5$	$8.5{+}e5$	$7.0{+}e5$	$1.2{+}e6$	$1.0{+}e6$	$6.4{+}e5$	$8.4{+}e5$	$7.9{+}e5$	$9.5{+}e5$	$1.2{+}e6$	$1.6{+}e6$	$1.1{+}e6$

**Fig. 4:**
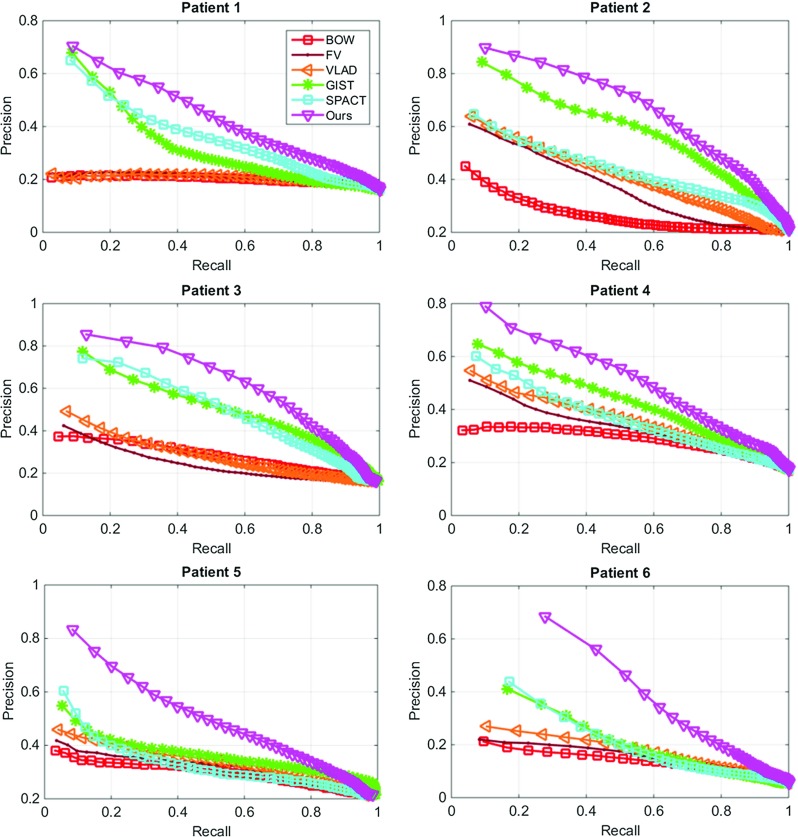
Precision-recall curves of descriptor evaluation on patient-specific experiments

## Experiments and results

### Dataset and protocol 

We implemented our framework on an HP workstation with an Intel $\times$5650 CPU and 24GB RAM, using Matlab and C++. Performance evaluation of our framework was conducted on in vivo data. We collected 13 video sequences (${\approx }17,700$ images) from standard GI endoscopic examinations on six patients. Two videos were collected in successive endoscopies for each of Patients 1–5. Three videos were collected for Patient 6 in serial examinations with time intervals of 3–4 months apart. Standard Olympus endoscope systems were used for video recording in $720\times 576$-pixel size, and the black borders in the images were removed before applying our framework. The NBI mode was turned on during data acquisition for image enhancement.

**Table 2:** Mean average precisions for retrieval performance. Our descriptor is compared to a range of popular descriptors

Methods	BOW	FV	VLAD	GIST	SPACT	Ours
Patient 1	0.227	0.233	0.234	0.387	0.411	0.488
Patient 2	0.307	0.418	0.468	0.636	0.477	0.722
Patient 3	0.321	0.290	0.338	0.576	0.595	0.705
Patient 4	0.331	0.391	0.425	0.495	0.412	0.573
Patient 5	0.341	0.361	0.390	0.415	0.389	0.556
Patient 6	0.201	0.203	0.242	0.345	0.315	0.547

**Fig. 5:**
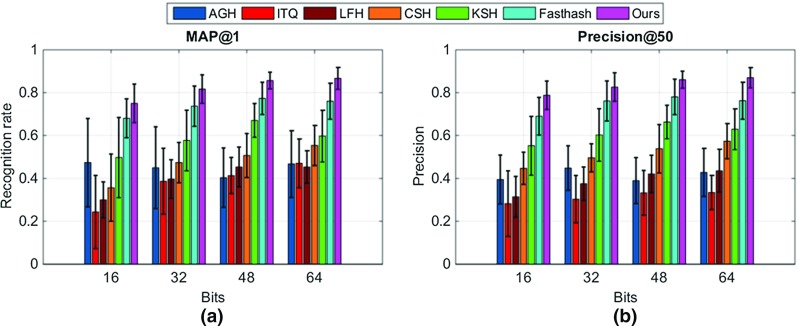
Evaluation of different binary code lengths. **a** Means and standard deviations of recognition rates, defined as mean average precisions with top retrievals (MAP@1); **b** means and standard deviations of precision values with top 50 retrievals (P@50)

**Fig. 6:**
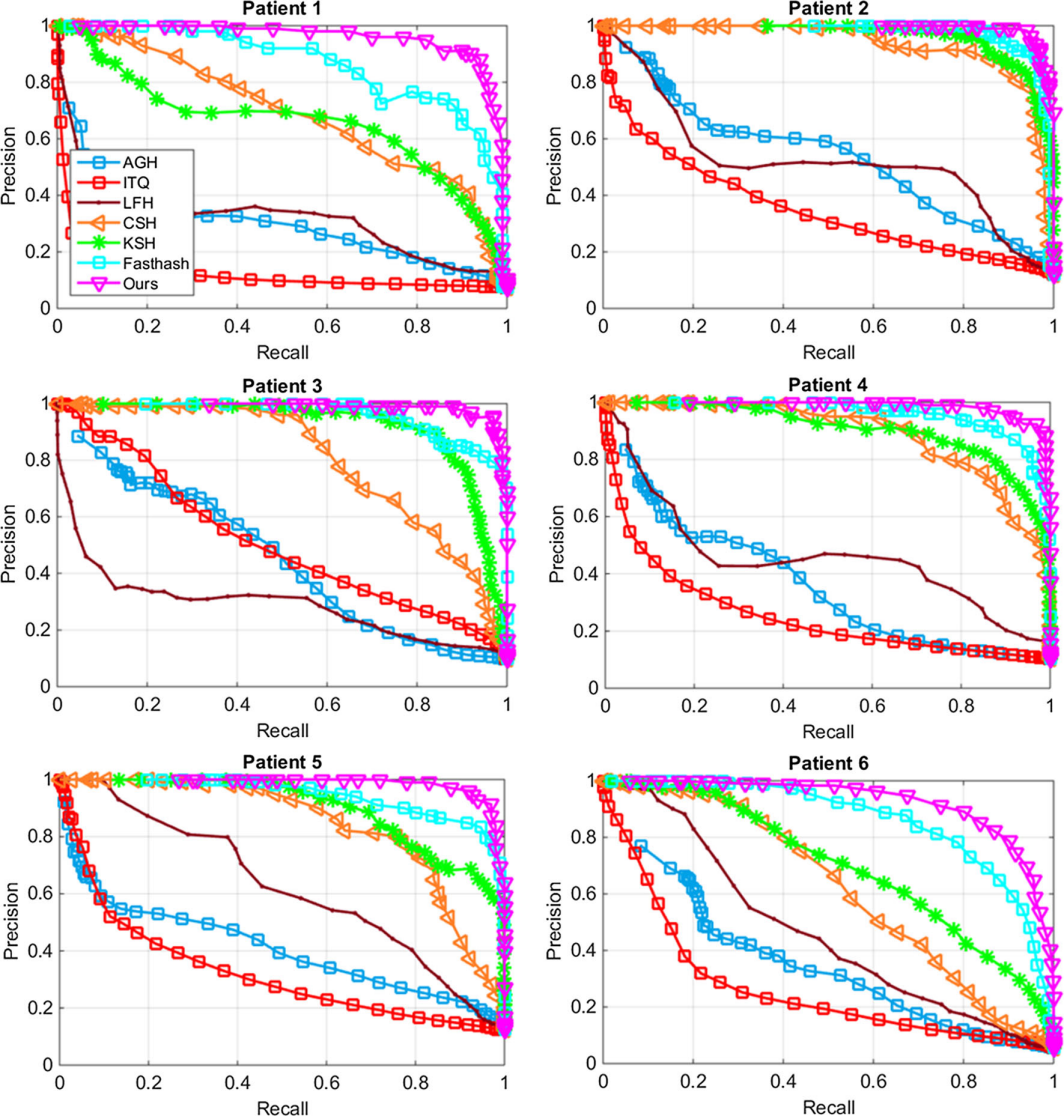
Precision-recall curves of framework evaluation on patient-specific experiments. Our hashing scheme is compared to state-of-the-art approaches on 64-bit binary code

In this work, we consider retargeting for patient-specific data; therefore, the random forests mapping function needs to be trained separately for each patient.. Leave-one-video-out validation was performed on the patients individually, which results in 16 experiments in total. For each experiment, one video was used as $\mathcal {O}_1$ for binary code inference and mapping function learning, and the other video was used as $\mathcal {O}_2$ for testing with randomly selected 50 query images. For obtaining the ground truth, intensity-based K-means clustering was performed on $\mathcal {O}_1$ and $\mathcal {O}_2$, resulting 10-34 clusters depending on video lengths. The clusters in $\mathcal {O}_1$ and $\mathcal {O}_2$ are then matched side-by-side manually by an expert, which generates the scene labels for the testing images (by checking their belonged clusters). The value of K is empirically determined according to the number of images contained in each video sequence. Our experiments did not focus on evaluating the sensitivity of the value of K on the framework performance, because this is a parameter which would be defined according to the particular clinical task. For example, for precise retargeting by trading off recall, then a larger value of K would be used to divided the sequence into a greater number of distinct clusters. It took around ten minutes for the expert to review obtained clusters for each video. We provide in Table 1 the details of the clustered video dataset, and their inter-cluster variances (ICV) [25].

### Evaluation metrics

We employed precision-recall analysis in evaluating both our descriptor and hashing framework. Let us now consider the top *U* image attempts retrieved from $\mathcal {O}_1$ relevant to a query in $\mathcal {O}_2$. A retrieval attempt is marked as true positive (TP) if it has the same scene label as the query, and false positive (FP), otherwise. Precision is then defined as the fraction of retrievals that are TP: $P = \frac{\# TP}{U}$, and recall is calculated as $R = \frac{\# TP}{V}$, where *V* is the number of all relevant images to the query. Mean average precision (MAP) is also used in evaluation as an indicative measure for image retrieval. When *Q* queries are tested and *U* retrievals are made, the MAP is obtained as11where  represents the precision of *q*-th query with the top *u* retrieval attempts. In addition, we also define  as the mean recognition rate, which represents the reliability of a system for returning its top ranked result.

### Descriptor evaluation

The proposed descriptor in this work has been validated against several popular image descriptors, including the GIST [26] descriptor based on wavelet responses, and a SPACT descriptor [17] based on pixel comparisons. We also compared to the BOW descriptor [13] using SIFT features. Furthermore, the popular variants of BOW, including Fisher vector (FV) [27] and VLAD [28] are also added into this comparison. For GIST, we performed $4\times 4$ partitioning on the image, and each partition was convolved with Gabor filters of 4 scales and 8 orientations, which results in a 512-*d* descriptor. We also followed [17] to implement a 1240-*d* SPACT descriptor using pixel-based census transform. For BOW, we created a dictionary that contains 10,000 words by sampling the SIFT features from the GI video sequences. For FV and VLAD, we used the publically available code to obtain 8192-*d* descriptors, followed by extracting their principal components to finally derive 256-*d* descriptors.

We present in Fig. 4 the precision-recall curves of our descriptor compared to the others. These curves are generated by varying the value of *U* and presented for patient-specific experiments. It can been seen that our descriptor outperforms the others in all experiments. We can also observe that the BOW approach has provided inferior results to the others due to the dependence on consistent keypoint detection, which is not reliable with long-term appearance changes on tissue surface (Patient 6 in Fig. 4). This also makes other variants of BOW including FV and VLAD generate similar results. Table 2 shows the MAP measures with our descriptor presenting the highest values in all experiments. Although GIST provides robustness to deformation, it lacks in encoding of the local texture details. The multi-level spatial pooling scheme in our descriptor ensures the similarities can be obtained across a range of scales. Our descriptor also outperforms the SPACT descriptor for the regional comparisons, due to better tolerance to illumination changes and camera translation.

**Table 3:** Mean average precisions for retrieval performance. Our entire framework is compared to state-of-the-art hashing schemes (using 64-bit) and a previous retargeting approach

Methods	EVM	AGH	ITQ	LFH	CSH	KSH	Fasthash	Ours
Patient 1	0.238	0.340	0.145	0.460	0.709	0.686	0.802	0.920
Patient 2	0.304	0.579	0.408	0.642	0.899	0.921	0.925	0.956
Patient 3	0.248	0.501	0.567	0.458	0.799	0.903	0.911	0.969
Patient 4	0.274	0.388	0.289	0.585	0.852	0.889	0.923	0.957
Patient 5	0.396	0.435	0.342	0.715	0.835	0.883	0.896	0.952
Patient 6	0.273	0.393	0.298	0.500	0.641	0.669	0.812	0.895

**Fig. 7:**
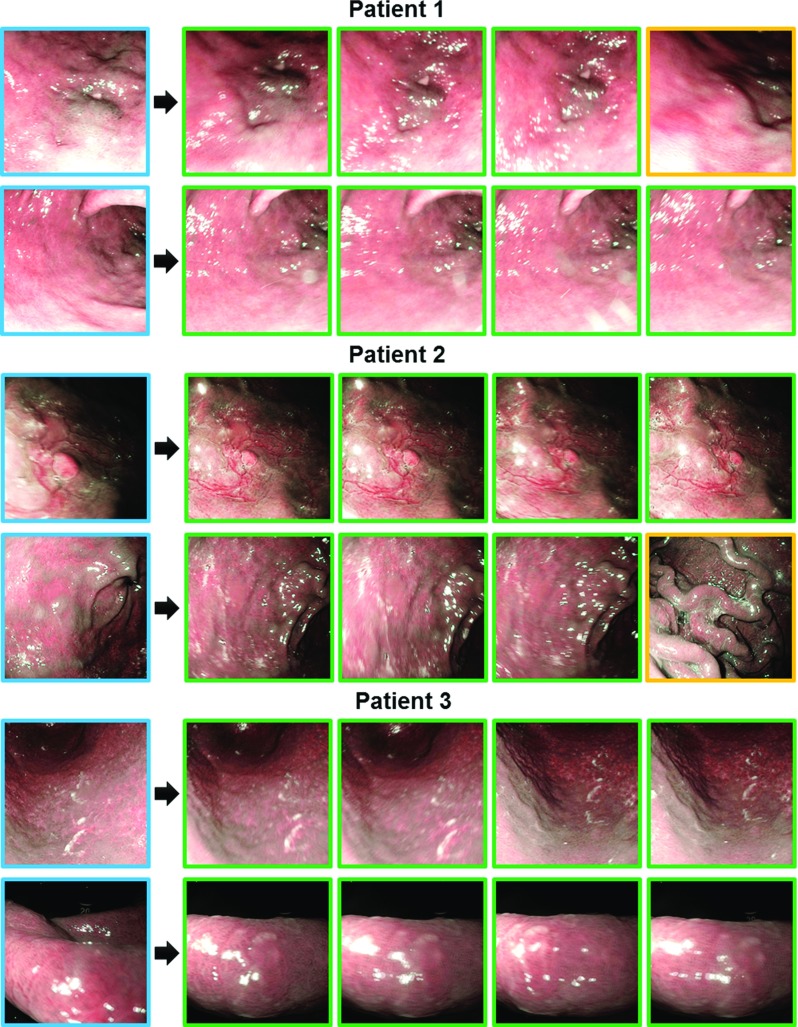
Example results for Patients 1–3. Top ranked retrievals based on Hamming distances, with *blue*-, *green*-, and *yellow*-border images being queries for retargeting, correct retargeting, and incorrect retargeting results, respectively

**Fig. 8:**
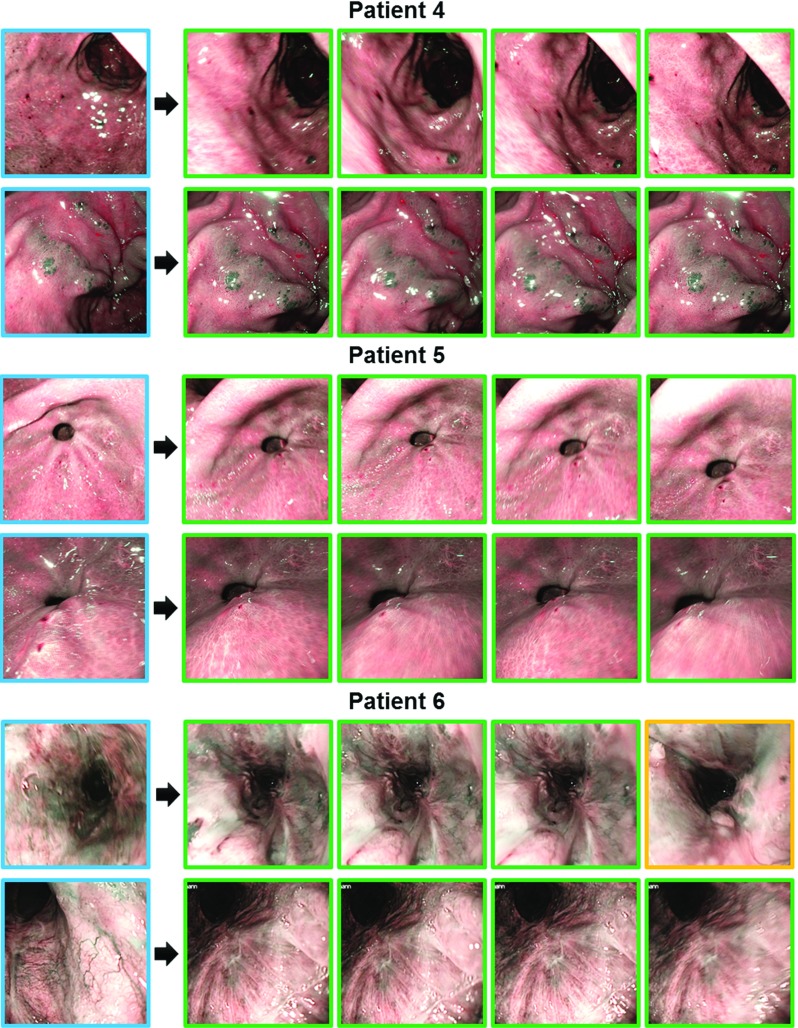
Example results for Patients 4–6. Top ranked retrievals based on Hamming distances, with *blue*-, *green*-, and *yellow*-border images being queries for retargeting, correct retargeting and incorrect retargeting results, respectively

### Framework evaluation

For evaluating the entire framework (after hashing), we compared to a range of state-of-the-art hashing approaches. These include hashing via iterative quantization (ITQ) [23], anchor graph hashing (AGH) [21], kernalised supervised hashing (KSH) [22], and Fasthash [24]. In addition, comparisons to two more recently proposed hashing approaches including hashing with latent factor models (LFH) [29] and column sampling based hashing (CSH) [30] were also performed. We also compared our framework to a relevant retargeting approach named endoscopic video manifolds (EVM) [8]. Each random forest for the mapping function in our framework contained 100 trees, with a maximum depth of 10 for each tree. We provide in Fig. 5a the recognition rates of all hashing-based approaches on different lengths ($m=\lbrace 16,32,48, 64\rbrace$) of binary codes, where our hashing scheme provides the highest recognition rates $\lbrace 0.75, 0.82, 0.86, 0.87\rbrace$. We also present the precisions with 50 top retrievals on all lengths in Fig. 5b, showing ours performs the best with $\lbrace 0.79,0.83,0.86,0.87\rbrace$. It is evident that 64-bit binary codes present the best performance, and we therefore use this length for the remaining evaluation.

The precision-recall curves of patient-specific experiments for all hashing-based approaches (64-bit) are provided in Fig. 6 with their associated MAP measures reported in Table 3. We observe from this table that after hashing, the retargeting performance has improved over the original descriptor (Table 2). In addition, our hashing scheme outperforms other alternatives, providing graceful falloffs in precision-recall, as well as the highest MAPs. The employed two-step hashing scheme provides flexibility in using independent classifiers for learning the mapping function, thus achieving more powerful discrimination than the approaches in [21–23, 29]. We also find that linear classifiers used in [30] are less discriminative than our classifiers, and boosted trees (Fasthash [24]) tend to overfit the training dataset, presenting lower MAP scores to our random forest-based hashing. It is worth noting the comparison to the EVM method, from which we notice that EVM generates inferior results to ours, and its performance on a similar dataset in our experiments is poorer than the one reported in [8]. This is because in our work, we use two different sequences from training and testing, yielding a realistic retargeting scenario, whilst in their studies training and testing data are from the same sequence. Finally, we present example retargeting results of our framework in Figs. 7 and 8.

Run-time speed is an important factor in using computer vision techniques for endoscopic interventions. A vision algorithm is usually required be real time such that a regular clinical procedure would not be interrupted. Our framework currently performs retargeting of one query within 19*ms*, which includes extracting the image descriptor, mapping into a binary code, and computing Hamming distances. Whilst the querying time using the original descriptor is around 490*ms*, the run-time speed improved by hashing meets the requirements of real-time capability.

### Discussion on limitation and use

It is worth noting the limitation of the current dataset, in which there are three videos collected from one patient within long-term intervals, and the other videos were collected from patients with serial endoscopies during one examination. Nevertheless, our experimental protocol follows realistic scenarios in surveillance endoscopy that only videos collected in ‘previous examinations’ are known, and used for subsequent examinations of the same patients. Our vision-based retargeting framework in this work provides relevant images of a query image of the same patient and does not provide the depth information of the endoscopic cameras [3] or specific locations (within images) of optical biopsies [4]; however, it can be used as an additional function to assist endoscopists by performing image retrieval for patient-specific data collected in serial examinations.

## Conclusions and future work

We proposed in this paper an image retrieval framework for inter-examination retargeting in gastrointestinal endoscopy. An image descriptor was proposed to consider the global geometry of an endoscopic scene by pooling the regional information at multi-scale. The extracted image descriptors from a previous video sequence were compressed into short binary codes via hashing. To allow for retargeting of a query image in the current examination, we proposed a novel random forest-based mapping, which provides not only strong discrimination in learning the mapping function, but also real-time retargeting capabilities. We compared our framework to a range of popular descriptors and hashing-based approaches. Experiments were conducted on in vivo video data collected from six patients, demonstrating the consistent state-of-the-art performance provided by our descriptor and hashing.

Currently, the framework learns the mapping function using only one previous video sequence. As further videos could be collected for the same patient, our framework can be readily extended to learn the mapping using two or more previous video sequences, which could further improve the retargeting performance. In addition, future works would also involve performing hierarchical image matching for further speedup or employing convolutional neural networks as more training data become available.
